# As the Turkish Journal of Anaesthesiology and Reanimation Leaves Its 50^th^ Anniversary Behind

**DOI:** 10.4274/TJAR.2024.231518

**Published:** 2024-02-28

**Authors:** Hatice Türe, Özge Köner, Ezgi Aytaç, Aslı Dönmez, Mois Bahar

**Affiliations:** 1 Yeditepe University Faculty of Medicine, Department of Anaesthesiology and Reanimation İstanbul, Turkey; 2 Etlik City Hospital, Clinic of Anaesthesiology and Reanimation, Ankara, Turkey; 3 İstanbul University-Cerrahpaşa, Cerrahpaşa Faculty of Medicine, Department of Anaesthesiology and Reanimation (Professor Emeritus), İstanbul, Turkey

**Keywords:** Anaesthesiology and Reanimation, History, History of the Turkish Journal of Anaesthesiology and Reanimation, History of TJAR, Turkish Journal of Anaesthesiology and Reanimation

## Abstract

The Turkish Journal of Anaesthesiology and Reanimation, established in 1972, is 50 years old now. The number of citations of the journal and the interest of national and international researchers are high. This success has been achieved by the editorial boards who have contributed to the journal since its establishment and the writers who have contributed to its development, and this success will continue to increase.

**Main Points**

• Scientific publications are essential in examining countries' scientific development levels. The Journal of the Turkish Society of Anaesthe­siology and Reanimation, established in 1972 has been publishing in the last 50 years.

• The number of citations of the journal and the interest of national and international researchers are high. This success has been achieved by the editorial boards who have contributed to the journal since its establishment and the writers who have contributed to its development, and this success will continue to increase.

## Introduction

Scientific publications are essential in examining countries’ scientific development levels.^1^ The Journal of the Turkish Society of Anaesthesiology and Reanimation, established in 1972 has been publishing in the last 50 years.

The Turkish Journal of Anaesthesiology and Reanimation (Turk J Anaesthesiol Reanim-TJAR) is a periodic scientific publication of the Turkish Society of Anaesthesiology and Reanimation. This editorially independent, unbiased, international journal periodically publishes peer-reviewed work in the anaesthesiology and reanimation field on a global scientific basis. The publication language is English. The journal is published bimonthly in February, April, June, August, October, and December.^2^

The improvement and progress made with the guidance of pioneer physicians of our field in the first hundred years of the Turkish Republic will pave the way and enlighten us in future centuries.

### Main Text

**Figure 1:**
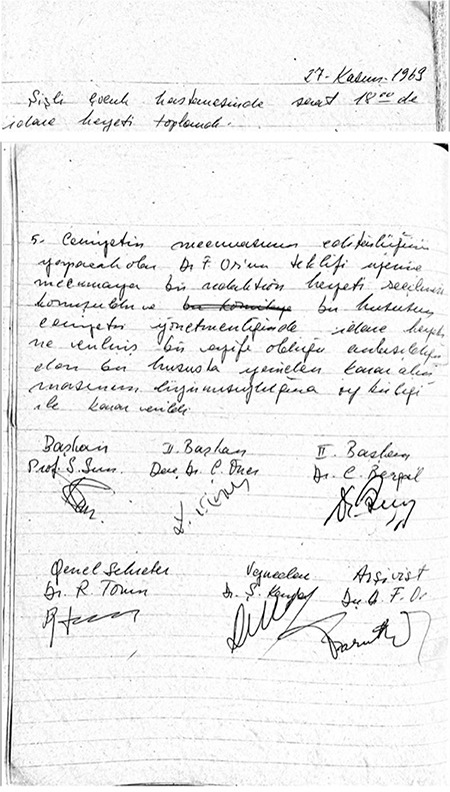
One of the attempts to publish a journal in 1969.

The publishing industry has come a long way, from clay tablets to audiobooks. Scientific publishing has a particular role in this long story. On January 5, 1665, French writer Denis de Sallo, Sieur de la Coudraye (1626 - May 14, 1669) published the first issue of the scientific journal, named “*Journal des sçavans*”. Over time, many scientific journals have been published with the developments in science and technology. With the branching out of medicine, anaesthesia has also developed as a main branch of surgery. In the 1950s, throughout the decade, the world continued its recovery from World War II, aided by the post-World War II economic expansion. During this period, anaesthesiologists began to organise worldwide, and associations of anaesthesia were established. The same historical developments have occurred in the Republic of Turkey. Anaesthesiologists Dr. Sadi Sun, Dr. Sabahat Kabaalioğlu and Dr. Cezmi Kınoğlu came together with Surgeons Dr. Şinasi Hakkı Erel and Dr. Fahri Arel to establish “Türk Anestezi Cemiyeti” (TAC) (*Turkish Community of Anaesthesia*) in November 12, 1956.^3^ Dr. Fahri Arel has become the first president of the “Turkish Community of Anaesthesia”. Two years later, on April 7, 1958, Sadi Sun (1922-1995) was elected president. Later, on January 1, 1969, this society will be named “Türk Anesteziyoloji ve Reanimasyon Cemiyeti (TARC) (*Turkish Community of Anaesthesiology and Reanimation*) parallel to the changes all over the world.^4^ There were many attempts to publish a journal in the first 16 years of the community’s establishment (Figure 1).

**Figure 2:**
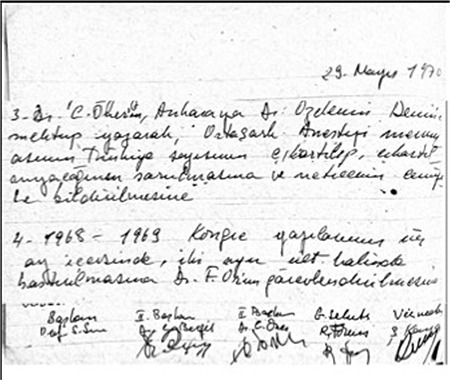
On 27 November 1970, board of the directors decided to publish the articles of the national congress and translate the journal of the Middle East Anaesthesia.

On 27 November 1970, board of the directors of the Turkish Society of Anaesthesiology and Reanimation decided to publish the articles of the national congress and translate the journal of the Middle East Anaesthesia (Figure 2).

**Figure 3:**
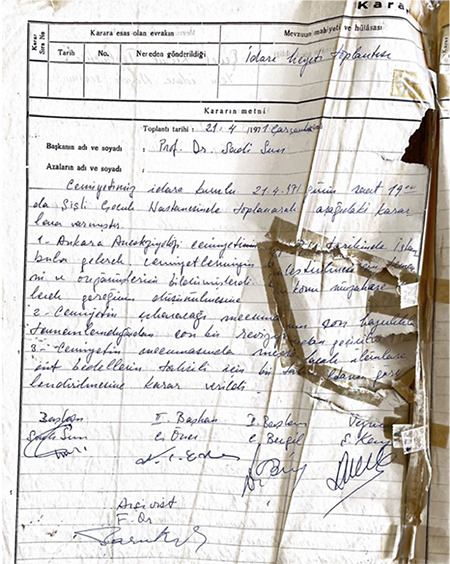
Historical note was written for final preparation of TJAR on April 21, 1971. TJAR, Turkish Journal of Anaesthesiology and Reanimation

On April 21, 1971, the board meeting of Turkish Society of Anaesthesiology and Reanimation was held in Şişli Etfal Children’s Hospital, and this historical note was written in the notes of the meeting; “the final preparations of the journal were completed” (Figure 3).

**Figure 4:**
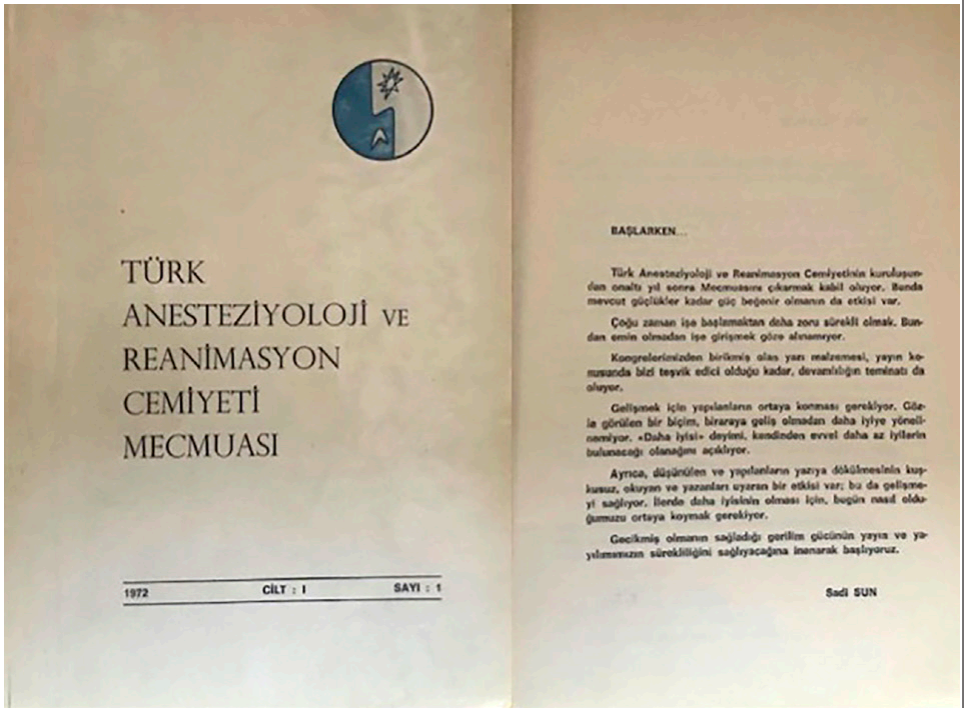
First issue of “The Journal of Turkish Community of Anaesthesiology and Reanimation“ in 1972.

Following these attempts in 1972, 16 years after the establishment of the *Turkish Community of Anaesthesiology and Reanimation*, “Türk Anesteziyoloji ve Reanimasyon Cemiyeti Mecmuası” (*The Journal of Turkish Community of Anaesthesiology and Reanimation)* has been published (Figure 4). During that period, Dr. Sadi Sun, one of the most important pioneers in anaesthesiology and reanimation in Turkey was assigned as the owner of the journal. From 1972 to 1985, Dr. Abdulkadir Erengül was assigned as editor-in-chief in the journal’s early years.

He collected and published the journal using the abstracts, conference contents and some articles and conferences of the previous year national congress. These journals were delivered to the members of society during the next congress. During these periods, the journal was published under challenging conditions. Annual volumes were created with several issues of the journal. They were archived in 12 books over 12 years; today, they are available in the society’s collection.

Following the social problems that took place in our country in 1980s, significant changes occurred in the law on associations, and after a 5-year silent period, important steps were taken to continue publishing the journal with more systematic and future-oriented projects.

**Figure 5:**
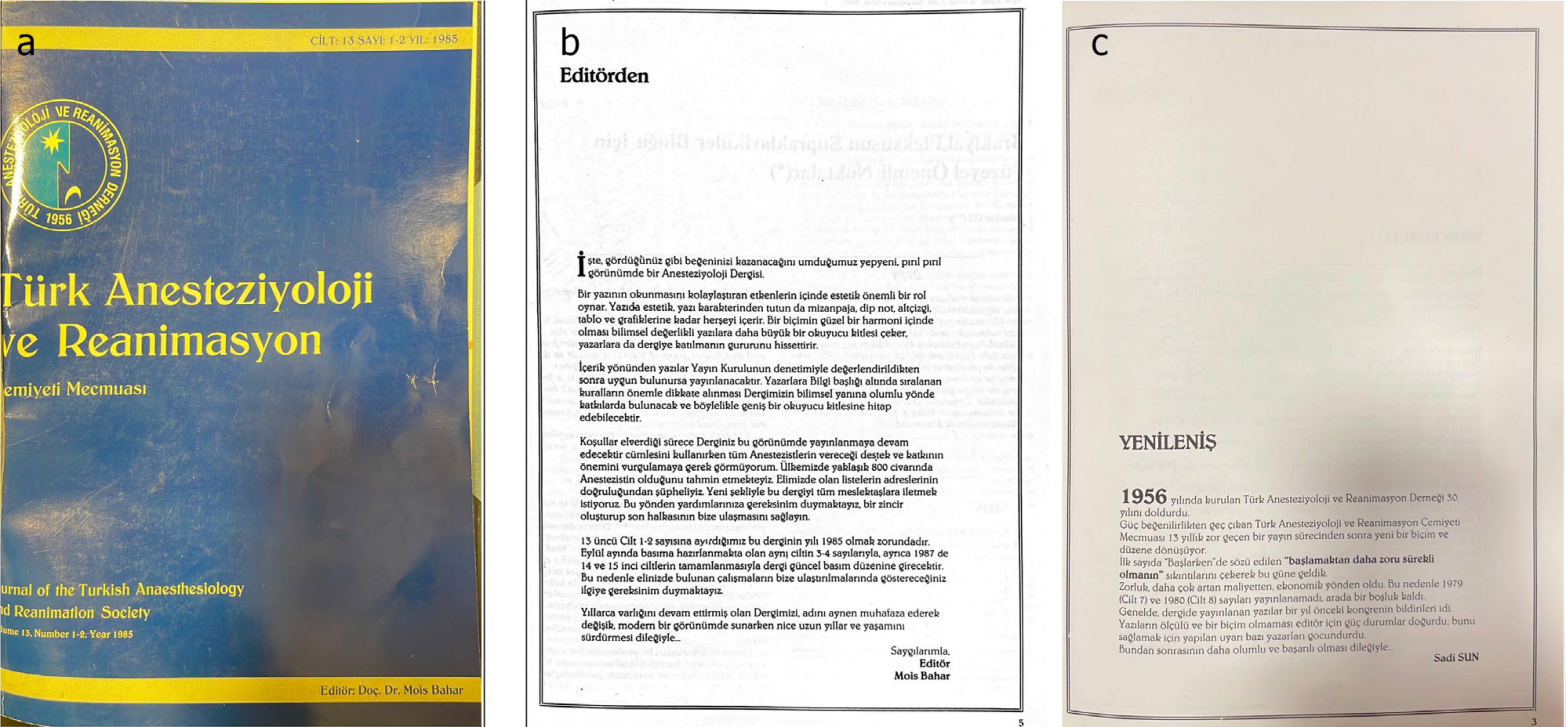
a) Cover Page of the 13^th^ volume of TJAR. b) Editorial article from Prof. Dr. Mois Bahar in 1985. c) Prof. Dr. Sadi Sun discussed and narrated future of the TJAR. TJAR, Turkish Journal of Anaesthesiology and Reanimation

Dr. Sadi Sun has been the president of TARC for 34 years (1958-1994), he assigned Dr. Mois Bahar as editor-in-chief from 1985 until 2002. In the editorial article in the first issue of the 13^th^ volume, Dr. Sadi Sun discussed and narrated their plan (Figure 5).

During this period, the standards and content of the journal have improved to the international level of those years. Articles which will be published in the journal were started to be evaluated by two referees. First, the journal began to be published 4 times a year, then 6, 8 and 10 times a year. Consequently, two supplements were published every available year, and Dr. Bahar professionally collected all journals from the first 17 years.

**Figure 6:**
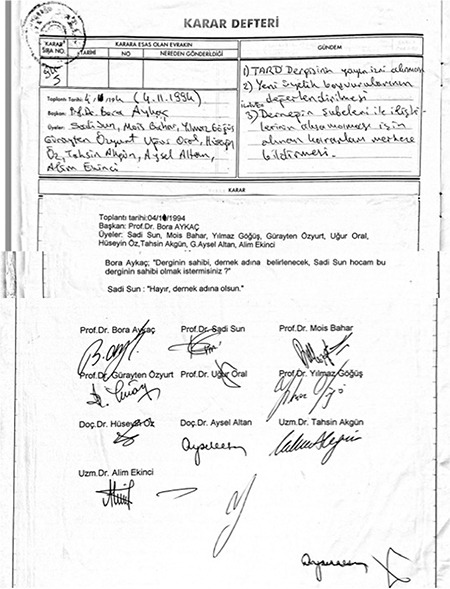
From this date on, president of the TARD began to serve as the owner of the TJAR. TARD, Turkish Anesthesiology and Reanimation Association; TJAR, Turkish Journal of Anaesthesiology and Reanimation

Another milestone decision can be read in society’s history with the president and members on 04.10.1994. Dr. Bora Aykaç (president), Dr. Sadi Sun, Dr. Mois Bahar, Dr. Yılmaz Göğüş, Dr. Gürayten Özyurt, Dr. Uğur Oral, Dr. Hüseyin Öz, Dr. Tahsin Akgün, Dr. G. Aysel Altan, and Dr. Alim Ekinci as a member of TARC have been discussed the future of TJAR (Figure 6).

In this meeting, parallel to the country’s regulations changes, it is decided to transfer the publication and legal rights of the journal from the legal entity to the corporate identity of the community. From this date on, president of the TARD began to serve as the owner of the TJAR.

While Dr. Mois Bahar served as an editor-in chief of the TJAR for 17 years, Dr. Sadi Sun (1985-1994), Dr. Bora Aykaç (1994-1996), Dr. Kutay Akpir (1996-1998), Dr. Uğur Oral (1998-2000), Dr. Oya Kutlay (2000-2002) and Dr. Filiz Tüzüner (2002-2003) served as the president of society.

**Figure 7:**
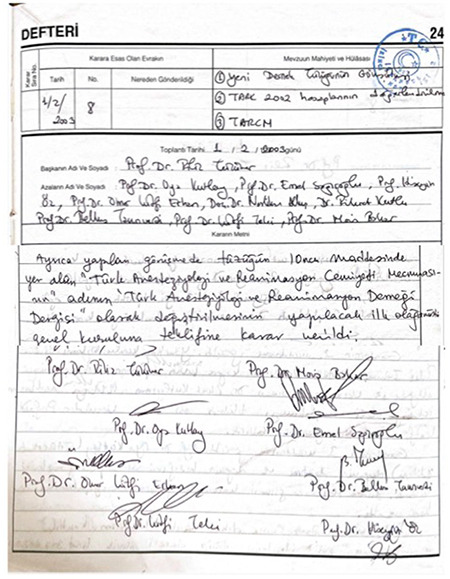
The “community” name was changed to the “society”, and the magazine (mecmua) name was changed to the ”journal” in February 2003.

Following these period, on 1 February 2003, the board of directors of TARC approved the historical decision, and the “community” name was changed to the “society”, and the magazine (Mecmua) name was changed to the ”journal” while Dr. Filiz Tüzüner was president of the society, and Dr. Oya Kutlay was editor-in-chief (Figure 7). In order to adapt the journal into the international scientific world, the English name of the journal started to be used. Then the name of the journal was reorganized, and “Reanimation” word was changed to the “Intensive Care” by the decision of the editorial board. However, this important and current change has not been last long. During these years, manuscripts have been initiated to transition to the e-journal format in order to ensure easy accessibility, increase the reading rate, increase referencing and speed up the Author-Editor-Reviewer process. Also, the journal was available to all members free of charge.

In June 2003, the journal was scanned by TÜBİTAK ULAKBİM, a nationally significant database for scientific publication. Another important effort was initiated to include the journal in Index Medicus; however, it failed. During these years, an important step was taken for the future of the journal and an international advisory board was established.

While Dr. Ali Reşat Moral was president of the society, and Dr. Filiz Tüzüner was editor-in-chief, an important step was taken for the future of the journal and an international advisory board was established.

Following millenium, while Dr. Ülkü Aypar was president of the TARD, and Dr. M. Erdal Güzeldemir was editor-in-chief, the journal has become available online. In order to expand the journal’s effect zone and increase its scientific level to the international area, the publication language has been changed from Turkish to English.^4^ In the following years, editors and editorial boards worked to ensure that the journal was included in internationally accepted indexes. The journal has been scanned in INDEX MEDICUS COPERNICUS since February 2009, in EBSCO since August 2009, in ICMJE since June 2009, and in BRITISH LIBRARY (since 1986).

With these changes over the years, the journal has been progressively prepared for inclusion in an international indexing platform. During Dr. Melek Tulunay’s tenure as editor-in chief and Dr. Şükran Şahin’s presidency of the society (2010-2012), these structural changes continued, and the necessary requirements for the journal to be included in international indexes were fulfilled.

Over the years, technological changes have been reflected to the journal, and official website was activated in January 2012. During these period, Dr. Güner Kaya was the president of society and, Dr. Yalım Dikmen was editor-in-chief of TJAR. In September 2012, in order to improve the impact factor of the journal, and in accordance with the regulatory rules of the international scientific indexing databases, the name of the “society” was removed from the name of the “Turkish Journal of Anaesthesiology and Reanimation” by the decision of the editorial board.^4^

**Figure 8:**
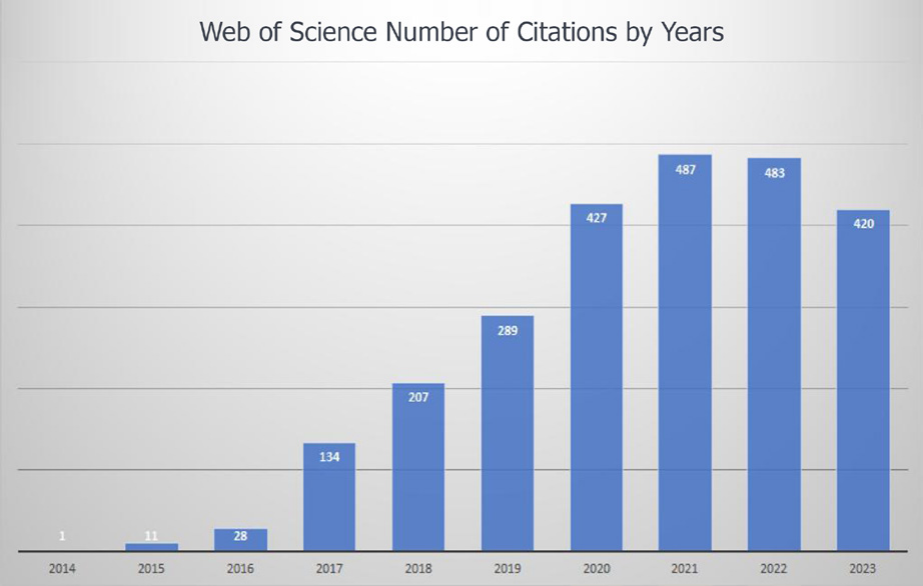
Citations per year (Web of Science).

2013 was a turning point in terms of the results of many years of work, then the journal met the indexing criteria by PubMed Central and the Web of Science-Emerging Sources Citation Index (ESCI). Dr. Yalım Dikmen was editor-in chief, and Dr. Güner Kaya was the president of TARD at that time. After this period, the number and scientific level of submitted manuscripts increased significantly (Figure 8).

Between 2016 to 2018, while Dr. N. Mert Şentürk was served as the editor-in chief of the TJAR and, Dr. Hülya Bilgin as the president of TARD, “debate articles” and “forums” from experts were published to enhance the journal’s impact factor. These articles were widely referenced across various platforms. Additionally, one of these articles was submitted by the World Federation of Societies of Anesthesiologists as a reference to the World Health Organization. By the end of 2018, significant increases in the impact factor were observed, leading to the journal being ranked as the 5^th^ best journal in ESCI (0.637).

Later on, accessibility and organizational capacity were expanded during Dr. Yalım Dikmen’s tenure as editor-in chief again. Subsequently, Dr. Ömer Kurtipek (2018-2021) and then Dr. Meral Kanbak (2021-2023) served as the president of society.

Article Processing Charges (APCs) was started to charge to authors during the publication process since January 2023, while Dr. Aslı Dönmez was editor-in chief of TJAR, and Dr. Ali Fuat Erdem was the president of the society. In January 2024, Dr. Zekeriyya Alanoğlu assigned as editor-in-chief of TJAR following Dr. Dönmez.

Today, it is published bimonthly in February, April, June, August, October, and December, and indexed by national and international databases such as PubMed Central, ESCI, Scopus, DOAJ, TÜBİTAK ULAKBİM-TR Index, China National Knowledge Infrastructure, EMBASE, EmCare, CINAHL, ProQuest ve Gale. Recently, social media has become an essential tool in the world of journals. It allows journals to promote their work and helps them connect with their readers and stay up to date with the latest research in their field. TJAR has social media accounts on instagram (@turkishjar) (2022), facebook (Tjar-Tjar) (2022), and X platform (j_turkish) (2019).

**Figure 9:**
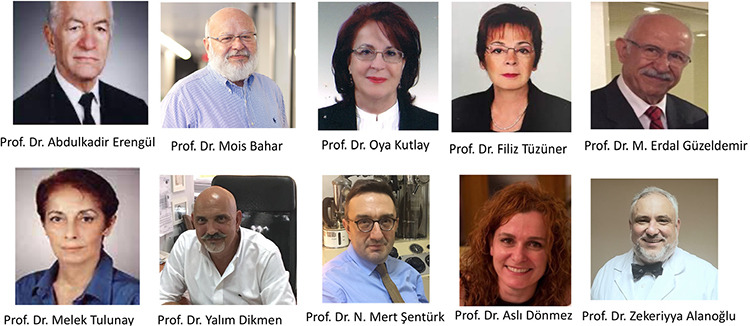
Editor in-Chiefs of the Turkish Journal of Anaesthesiology and Reanimation since its establishment in 1972.

As we celebrate the first 100^th^ anniversary of the Republic of Turkey, Turkish Journal of Anaesthesiology and Reanimation is a well-known journal of high scientific quality which results from outstanding efforts of its Editor-in-chiefs, editorial boards, reviewers and authors (Table 1, Figure 9).

## Conclusion

The Turkish Journal of Anaesthesiology and Reanimation (TJAR) is a peer-reviewed open access journal that meets high quality standards by exercising peer review and editorial quality control. This success has been achieved by the editorial boards who have contributed to the journal since its establishment and the writers who have contributed to its development, and this success will continue to increase. We wish many productive 50 years to TJAR to contribute to the scientific world.

## Figures and Tables

**Table 1:**
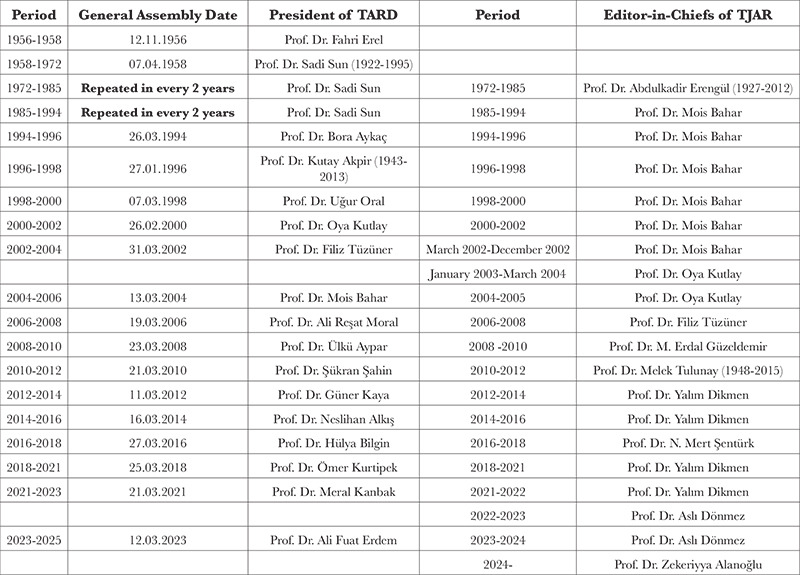
Editor-in-Chiefs of the TJAR
